# ﻿Redescription of a rarely encountered species *Travisachinensis* Grube, 1869 (Annelida, Travisiidae), including a description of a new species of *Travisa* from Amoy, China

**DOI:** 10.3897/zookeys.1128.90020

**Published:** 2022-11-04

**Authors:** Deyuan Yang, Xuwen Wu, Zhi Wang, Xiaoyu Zhao, Jiangshiou Hwang, Lizhe Cai

**Affiliations:** 1 Institute of Marine Biology, National Taiwan Ocean University, Keelung 20224, Taiwan National Taiwan Ocean University Keelung Taiwan; 2 College of the Environment and Ecology, Xiamen University, Xiamen 361102, Fujian, China Xiamen University Xiamen China; 3 Institute of Oceanology, Chinese Academy of Sciences, Qingdao 266071, China Institute of Oceanology, Chinese Academy of Sciences Qingdao China; 4 State Key Laboratory of Marine Environmental Science, College of Ocean and Earth Sciences, Xiamen University, Xiamen 361102, China National Taiwan Ocean University Keelung Taiwan; 5 Center of Excellence for Ocean Engineering, National Taiwan Ocean University, Keelung 20224, Taiwan Xiamen University Xiamen China; 6 Center of Excellence for the Oceans, National Taiwan Ocean University, Keelung 20224, Taiwan Institute of Oceanology, Chinese Academy of Sciences Qingdao China

**Keywords:** Morphology, phylogeny, Polychaeta, stinkworms, taxonomy

## Abstract

The original description of *Travisiachinensis* Grube, 1869 was incomplete, leading to confusion with other species. To clarify the status of this species, we provide a redescription of, and remarks on, *T.chinensis* based on an examination of the type specimen. We also describe *Travisiaamoyanus***sp. nov.**, collected from Xiamen (Amoy), China, and originally identified as *T.chinensis* by Monro (1934). The new species can be distinguished from its congeners by a combination of the following characters: the total number of segments (34 or 35) and chaetigers (33 or 34), parapodial lappets first from chaetiger 15, and a pygidium with a large ventral triangular cirrus and about six encircling lateral cirri. Genetic distances and phylogenetic analyses based on the mitochondrial (*16S rRNA*) and nuclear (*18S rRNA*) genes support the identity of the new species.

## ﻿Introduction

Currently, the polychaete family Travisiidae Hartmann-Schröder, 1971 contains a single genus, *Travisia* Johnston, 1840, which includes 37 recognized species (Read and Fauchald 2022). *Travisia* is easily recognized by its noticeably fetid odor which makes them known as stinkworms. Because their external features are very simple, the taxonomic characters are mainly based on quantitative morphological characters, such as the total number of segments, chaetigers, branchiae, and pygidial lobes (Dauvin and Bellan 1994; Rizzo and Salazar-Vallejo 2020). Although some reviews (Dauvin and Bellan 1994; Augener 1922) and regional studies (e.g., Hartman 1969; Yang and Sun 1988; Maciolek and Blake 2006) had been conducted on *Travisia*, there are still many species with incomplete descriptions, and species boundaries need to be re-evaluated.

*Travisiachinensis* Grube, 1869 was originally described based on a single specimen collected from Chinese waters, but the exact type locality was not given. Later, Augener (1922) redescribed *T.chinensis* based on the type material and considered *Travisiaolens* Ehlers, 1879, *T.kerguelensis* McIntosh, 1885, and *T.chinensis* as the same species, based on their similar number of segments. Unfortunately, none of these authors provided illustrations, and some main characters (e.g., the number of branchiae, and the position and shape of parapodia lappets) were not clearly described. Since these early descriptions, Monro (1934) identified a specimen collected from Amoy (Xiamen) as *T.chinensis* based on Augener’s (1922) statement. However, the main characters of *T.chinensis* sensu Monro, 1934 are not consistent with Grube and Augener’s descriptions, mainly differing in the number of chaetigers (33 or 34 vs 29) and the number of segments (34 or 35 vs 30). *Travisiachinensis* has not been recorded anywhere since its original description (Dauvin and Bellan 1994) and has rarely been compared with other *Travisia* species by subsequent authors. To clarify the taxonomic confusion, we examined the holotype of *T.chinensis* (ZMB 0629) deposited in Zoological Museum, Berlin (ZMB) with the help of Dr Birger Neuhaus. Detailed descriptions of *T.chinensis* are provided and compared with the related species.

The first author examined all materials of *Travisia* deposited in the Marine Biological Museum (MBM) of the Chinese Academy of Sciences (IOCAS). Newly collected *Travisia* specimens revealed that the specimens from the coastal region of Xiamen agree well with *T.chinensis* sensu Monro (1934) in most morphological characters, such as body with 33 or 34 chaetigers and parapodial lappets present from chaetiger 15. In addition, both our materials and Monro’s specimen were collected from Amoy (Xiamen), southern East China Sea. In this study, we consider them as a new species to science, *Travisiaamoyanus* sp. nov.

Our study aims to provide redescriptions and comments on the rarely known *T.chinensis* Grube, 1869, as well as to erect a new species, *T.amoyanus* sp. nov., collected from Xiamen Fujian, China. To confirm the taxonomic status of the new species, we studied the morphology of the specimens and performed phylogenetic analyses based on partial sequences of *16S rRNA* and *18S rRNA* genes. We also provide the *28S rRNA* gene sequence of this new species.

## ﻿Materials and methods

### ﻿Specimen collection and morphological study

The type material of *T.chinensis* (holotype, ZMB 0629) was examined at the Zoological Museum, Berlin (ZMB) by Dr Birger Neuhaus in June 2020. Twenty-five specimens of the undescribed species were collected from the coastal regions of Xiamen and deposited in the Marine Biological Museum of the Chinese Academy of Sciences in the Institute of Oceanology in Qingdao, China. Sampling information of the examined specimens is summarized in the Suppl. material 1: table S1. Methyl green (MG) stain saturated in 80% ethanol was used to highlight the external morphological characters and characterize MG staining patterns following the methods of Maekawa and Hayashi (1999). Specimens were observed using a Zeiss Discovery V20 or Motic SMZ-168 stereomicroscope. Macrophotographs of whole animals were photographed using a Canon EOS 6D Mark II with a 100 mm macro lens or and Olympus E-M1 Mark II with a 60 mm lens with LED lighting. Micrographs were taken using an AxioCam 512 digital camera mounted on the microscope. All image stacks were obtained using Helicon Focus v. 7. To assess intraspecific variation of morphological characters, for each complete specimen, we measured the total length (TL), maximum width (at widest segments of the body), counted the total number of segments and chaetigers, the number of branchiae (including the starting and end segments of branchiae), and the starting segment of parapodia lateral lappets. A statistical analysis was carried out using Microsoft Excel. The morphological terminology follows Blake and Maciolek (2020). The definition of the total number of segments follows the recent proposal of Rizzo and Salazar-Vallejo (2020). The following abbreviations are used: Toc, total number of chaetigers; Tos, total number of segments; Mob, Maximum of branchiae; Sopl, start of parapodial lappets on chaetiger; Sob, start of branchiae; pr, prostomium; per, peristomium; nuO, nuchal organ; mo, mouth; br, branchiae; chaet, chaetiger; IntP, interramal pore; Pl, parapodia lateral lappet; np, nephridial pore; ntc, notochaetae; npc, neurochaetae; Py, pygidium; Vc, Pygidial ventralmost cirrus; MG, Methyl Green.

### ﻿DNA extraction, amplification, and sequencing

Genomic DNA was extracted from branchiae or tissue of ethanol-preserved specimens using the Qiagen DNeasy Blood and Tissue Kit, following the DNeasy Protocol provided in the manufacturer’s instructions. Two nuclear gene markers: 28S and 18S and one partial mtDNA gene marker 16S were amplified and sequenced. Polymerase chain reactions (PCR) for the 28S and 18S genes followed the protocols of Glover et al. (2016), and 16S gene followed the protocols described by Law et al. (2014) and Kobayashi and Kojima (2021). The primers and PCR annealing temperatures are summarised in Suppl. material 1: table S2. Polymerase chain reactions (PCR) were conducted in a total volume of 50 µl containing 25 µl PCR Mix (Dongsheng Biotech Co., Ltd, Guangdong, China), 2 µl forward- and reverse-primer each (10 µM), 2 ul template DNA, and 20 µl ddH20. All PCR reactions were performed in a Veriti 96-Well Thermal Cycler (Applied Biosystems, Thermo Fisher Scientific). The PCR products were electrophoresed on a 1.5% agarose gel, then Sanger sequencing was performed by Sangon Biotech (Shanghai) Co., Ltd. SeqMan v. 11.1.0 (DNAStar, WI, USA) was used to assess the forward and reverse DNA strands of each gene, then blasted in GenBank to check for potential contamination. The newly obtained sequences were submitted to GenBank (http://www.ncbi.nlm.nih.gov) in the National Centre for Biotechnology Information (**NCBI**).

### ﻿Phylogenetic analyses

For phylogenetic comparisons, we used all available *Travisia* sequences downloaded from GenBank and our newly obtained sequences. As outgroups, the sequences of *Ophelialimacine* (Rathke, 1843), *Scalibregmainflatum* Rathke, 1843, and *Polyphysiacrassa* (Örsted, 1843) were used. All sequences used in this study were listed in Suppl. material 1: table S3. The online version of MAFFT v. 7 (Katoh et al. 2019) was used to align multiple sequences of each marker, with default values except for the parameter “Adjust direction according to the initial sequence,” which was turned on. The subsequent phylogenetic analysis steps were performed in PhyloSuite v. 1.2.2 (Zhang et al. 2020) with the help of its plug-in programs (Gblocks, ModelFinder, PartionFinder2, IQ-TREE v. 1.6.8, and MrBayes v. 3.2.6). Gblocks v. 0.91b (Talavera and Castresana 2007) was used to remove ambiguously aligned sites. Three data sets (16S, 18S, and 16S+18S) were conducted for the phylogenetic analysis with maximum likelihood (ML) and Bayesian inference (BI) analysis. ModelFinder (Kalyaanamoorthy et al. 2017) and PartionFinder2 were used to select the best-fit model for single gene (16S or 18S) and the concatenated sequences (16S and 18S) using the Bayesian Information Criterion (BIC). ML was performed using IQ-TREE (Nguyen et al. 2015) based on 10000 ultrafast bootstrap replicates. BI was performed using MrBayes (Ronquist et al. 2012). Analyses were run for 10 million generations, in which the initial 25000 sampled data were discarded as burn-in. The resulting ML and Bayesian trees were visualized in iTOL (https://itol.embl.de/).

Nucleotide divergence (*p*-distance and Kimura2-parameter) over sequence pairs within and between species of *Travisia* were calculated in MEGA X (Kumar et al. 2018).

## ﻿Results

### ﻿Phylogenetic analyses

The results of phylogenetic analyses (ML and BI) based on partial 16S rDNA (417 bp), 18S rDNA (1671 bp), and their concatenated sequences (2088 bp), showed different topologies and support values, but analyses of ML and BI based on each dataset have the same topologies.

**Figure 1.**
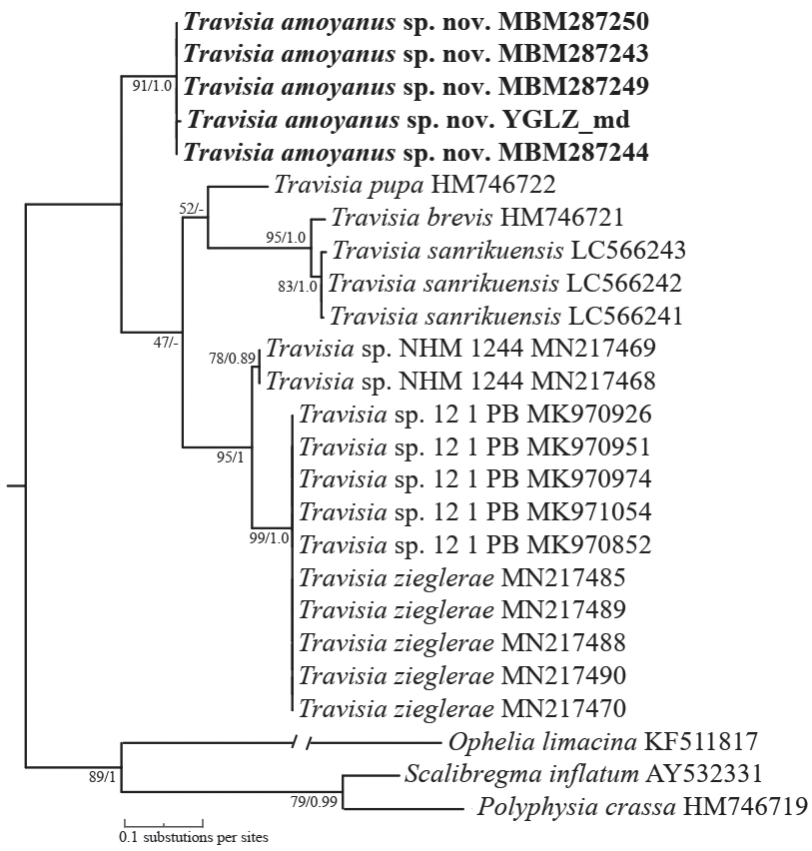
Maximum likelihood (ML) tree of Travisiidae based on 16S sequences under IM2+F+G4 model, GTR+G+F model was used for Bayesian inference (BI) analysis. Node support values based on 10000 ultrafast bootstraps from ML followed by posterior probability values from BI analyses.

**Figure 2.**
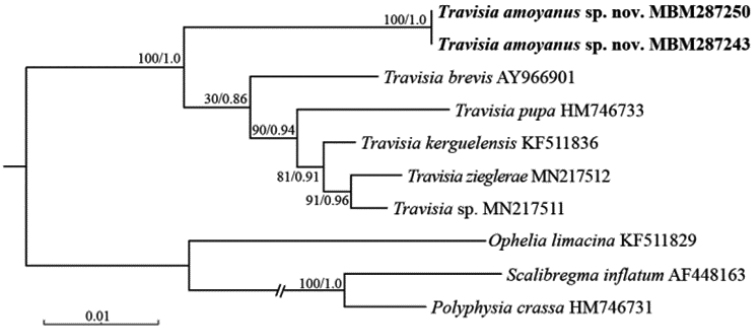
Maximum likelihood (ML) tree based on 18S rRNA gene sequences under TIM3+I+F model, K2P+I model for Bayesian inference (BI) analysis. Node support values based on 10000 ultrafast bootstraps from ML followed by posterior probability values from BI analyses.

Phylogenetic analysis based on 16S or 18S sequences indicated that *Travisiaamoyanus* sp. nov. was sister to all the other species of *Travisia* (BS = 89%, PP = 1.0 and BS = 100%, PP = 1.0, respectively; Figs 1, 2). However, phylogenetic analysis based on the concatenated sequences showed that *T.amoyanus* sp. nov. was sister to the clade consisting of *T.pupa* Moore, 1906, *T.kerguelensis* McIntosh, 1885, *T.zieglerae*Wiklund et al. 2019 and *Travisia* sp. (BS = 49%, PP = 0.53), and all the above species were sister to the clade consisting of *T.sanrikuensis* Kobayashi & Kojima, 2021 and *T.brevis* Moore, 1923 (Suppl. material 2). The pairwise genetic distances between *T.amoyanus* sp. nov. and the other species of *Travisia* ranged from 17.5% to 20.7% (Kimura2-parameter) and 15.4–17.9% (uncorrected *p*-distance) for 16S (Table 1), 3.4–3.9% (Kimura2-parameter) and 3.4–3.8% (uncorrected *p*-distance) for 18S (Suppl. material 1: table S4), while the intraspecific distance within *T.amoyanus* sp. nov. was 0.08% for 16S and 0.1% for 18S. Such large genetic distance ranges for 16S and 18S, much larger than for compared species, were sufficient to distinguish the *T.amoyanus* sp. nov. from those species.

**Table 1.** Pairwise distances using 16S within and among species of *Travisia*: values in the lower left corner were based on the Kimura2-parameter, and in the upper right corner were based on the *p*-distance model. Note: red numbers indicate the intraspecific genetic distance (*p*-distance and Kimura2-parameter show similar value).

	Species of *Travisia*	*N*	1	2	3	4	5	6	7
1	*T.amoyanus* sp. nov.	5	0.001	0.170	0.171	0.179	0.167	0.162	0.154
2	*T.zieglerae*	21	0.195	0.001	0.001	0.058	0.132	0.172	0.151
3	*Travisia* sp.	10	0.197	0.001	0.001	0.060	0.136	0.172	0.155
4	*Travisia* sp. NHM 1244	2	0.207	0.061	0.063	0.001	0.109	0.153	0.144
5	*T.pupa*	1	0.191	0.146	0.151	0.118	n/c	0.153	0.127
6	*T.sanrikuensis*	3	0.186	0.198	0.199	0.172	0.174	0.006	0.032
7	*T.brevis*	1	0.175	0.170	0.175	0.160	0.140	0.033	n/c

### ﻿Taxonomy

#### Family Travisiidae Hartmann-Schröder 1971

##### ﻿Genus Travisia

Taxon classificationAnimaliaPolychaetaTravisiidae

Johnston, 1840

E7ADC421-57F4-59C9-9E2A-10507956CE87

###### Type species.

*Travisiaforbesii* Johnston, 1840.

###### Diagnosis

**(based on Rizzo and Salazar-Vallejo 2020).** Body subfusiform or grub-like. No obvious ventral or lateral groove. Segments annulated, with integument papillated. Prostomium small, conical or truncate, with no eyes and prostomial processes. Nuchal organs present. Parapodia reduced to two fascicles of capillary chaetae, with no dorsal or ventral cirri. Parapodial lappets or lobes present above and below the fascicles of chaetae in some species. Branchiae present or absent. A series of interramal sensory organs or pores present between dorsal and ventral fascicles of chaetae. Nephridial pores present. Pygidium ovoid or cylindrical.

###### Remarks.

Three genera (*Dindymenides*, *Kesunis*, and *Travisia*) were included in the subfamily Travisiinae Hartmann-Schröder, 1971, and later *Dindymenides* and *Kesunis* were synonymized with *Travisia* by Dauvin and Bellan (1994). Blake and Maciolek (2020) elevated Travisiinae Hartmann-Schröder, 1971 to family Travisiidae, with *Travisia* as the only valid genus. However, the synonymization of these three genera by Dauvin and Bellan (1994) was only based on the morphological study and a molecular phylogenetic analysis has yet to have been done.

##### ﻿ Travisia chinensis

Taxon classificationAnimaliaPolychaetaTravisiidae

Grube, 1869

382E22A6-DB96-5A4A-9B03-221DE1EDECB9

Fig. 3A–C

Travisia chinensis Grube, 1869: 66; China Sea, North-western Pacific.
Travisia chinensis Augener, 1922: 38–40.

###### Diagnosis.

Body with 30 segments and 29 chaetigers. Branchiae cirriform from chaetiger 2, more than 25 pairs. Neuropodial lappet from chaetiger 16, notopodial lappet from chaetiger 19. Annulation pattern of segments: 1–15 triannulate, 16–26 biannulate, 26–30 uniannulate.

###### Material examined.

***Holotype*.**ZMB 0629, Chinese waters (“Chinesische Gewässer”), Coll. GRUBE.

###### Description.

**Figure 3.**
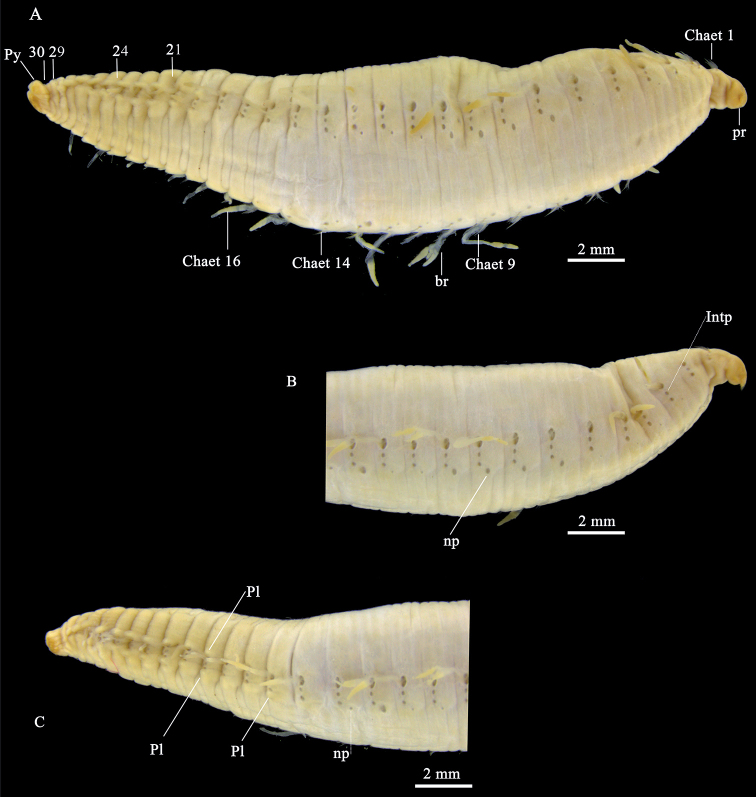
*Travisiachinensis* Grube, 1866 (holotype, ZMB 0629) **A** complete specimen in lateral view **B** anterior part in lateral view **C** posterior part in lateral view. Abbreviations: pr, prostomium; nuO, nuchal organ; mo, mouth; br, branchiae; chaet, chaetiger; IntP, interramal papilla; Pl, parapodia lateral lappet; np, nephridial pores; Py, pygidium.

Body fusiform. Whitish in alcohol. About 30 mm in length (Fig. 3A). Prostomium twisted, anteriorly pointed (Fig. 3B). The mouth between chaetiger 1 and chaetiger 2 (Fig. 3B). Branchiae cirriform, except one trifid present chaetiger 10 on the right side, more than 25 pairs, start on chaetigers 2 and to at least chaetigers 26 (Fig. 3A). Most branchiae shorter than body width.

Chaetigers 1–15 without parapodial lappets. Chaetiger 16 with a small neuropodial lappet, below the bundle of neurochaetae on the right side of the body (Fig. 3C). Notopodial lappet above the bundle of notochaetae starting on chaetiger 19. Notopodial and neuropodial lappets well developed from chaetiger 19, but missing on segments 29 and 30 (Fig. 3C). Nephridial pores from chaetigers 3–14, the first four and last four small, the remainder larger (Fig. 3A).

Neuropodial and notopodial chaetal rami well separated. Chaetae arising directly from body wall, with 29 chaetigers. All chaetae hair-like, smooth and without a fringe. Interramal pores from the first chaetigers segment to almost all segments except the last one segment. Segments 2–15 with three annulations, segments 16–26 with two annulations, last five segments with one annulation (Fig. 3A). Pygidium as long as last three segments, with about 10 indentations.

###### Remark.

The original description of *Travisiachinensis* was not detailed. Thus, it was seldom compared with the other *Travisia* species. According to the original description, *T.chinensis* has one trifid branchia, while most other *Travisia* species have cirriform branchiae, except for *T.arborifera* Fauvel, 1932 from Indian Ocean and *T.filamentosa* León-González, 1998 from California which were reported with strongly branched branchiae. Some researchers accepted that the trifid branchia might make *T.chinensis* a distinctive species (Kükenthal 1887; Fauvel 1932), while according to our observation, the trifid branchia is also present in a specimen of Travisiacf.pupa from the Yellow Sea (unpublished data), which is supposed to have only cirriform branchiae. Therefore, the presence of one bifid or trifid branchia might actually be an intraspecific variation and should not be regarded as a valid characteristic in distinguishing *Travisia* species.

*Travisiachinensis* (30 segments, 29 chaetigers) resembles the following six species in have a similar number of segments and chaetigers (29–31): *Travisiaamadoi* Elías et al., 2003, *Travisiaolens* Ehlers, 1897, *Travisiaaraciae* Rizzo & Salazar-Vallejo, 2020, *Travisiahobsonae* Santos, 1977, *Travisiabrevis* Moore, 1923, and *Travisiaforbesiiintermedia* Annenkova, 1937.

*Travisiachinensis* differs in the start of parapodial lappets (chaetiger 19) from *T.amadoi* (chaetiger 12), *T.araciae* (chaetiger 13), and *T.hobsonae* (chaetiger 1). *Travisiachinensis* differs from *T.brevis* in the following morphological characters: the number of branchiae (>25 pairs in *T.chinensis* vs 22 pairs in *T.brevis*); the shape of the prostomium (conical vs short blunt cone), and segments without parapodial lappets (last four segments vs last two segments).

*Travisiaforbesiiintermedia* and *T.olens* are not easily distinguished from *T.chinensis* more by lack of information. According to the original description, the former two lack exact data on the position of parapodial lappets, and a re-examination of the types of the two species is needed.

###### Type locality.

According to Salazar-Vallejo et al. (2014), the type locality was probably the coastal waters of Qingdao. Dauvin and Bellan (1994) also stated that the holotype was from the North-western Pacific. Until now, we have not found any other specimens of *T.chinensis* in the seas of China, based on the materials of MBM.

##### ﻿ Travisia amoyanus sp. nov.

Taxon classificationAnimaliaPolychaetaTravisiidae

3670D2AA-B384-52A4-A6D9-032C6BF6375E

https://zoobank.org/0211D399-6360-4932-9AB6-993260F8A26C

Figs 4A–O , 5A–H

Travisia chinensis Monro, 1934: 374, fig. 8.

###### Material examined.

***Holotype*.** Complete MBM287243: Xiamen, China, 24°27.14'N, 118°11.19'E, 24 July 2021, ethanol. ***Paratypes*.** One complete (MBM193597), two complete (MBM286089), Xiamen, China, 24°35.04'N, 118°10.09'E, 19 April 1963, formalin. Five complete (MBM286088), Xiamen, China, 24°26.30'N, 118°10.11'E, 2014–2016, formalin. Four complete (MBM286075), Xiamen, China, 24°30.49'N, 118°16.30'E, 2014–2016, formalin. One complete (MBM287244), one complete (MBM287245), one complete (MBM287248), one complete (MBM287249), one complete (MBM287250), same data as the holotype, ethanol one complete (MBM287246), one complete (MBM287247), same data as the holotype, formalin.

###### Diagnosis.

Prostomium pointed, conical. Body with 34 or 35 segments and 33 or 34 chaetigers. Branchiae cirriform from chaetiger 2 to chaetiger 28–32. Larger triangular lateral parapodia lobes or lappets well developed from chaetiger 15. Pygidium with a larger ventral triangular cirrus and about six lateral cirri around.

###### Description.

**Figure 4.**
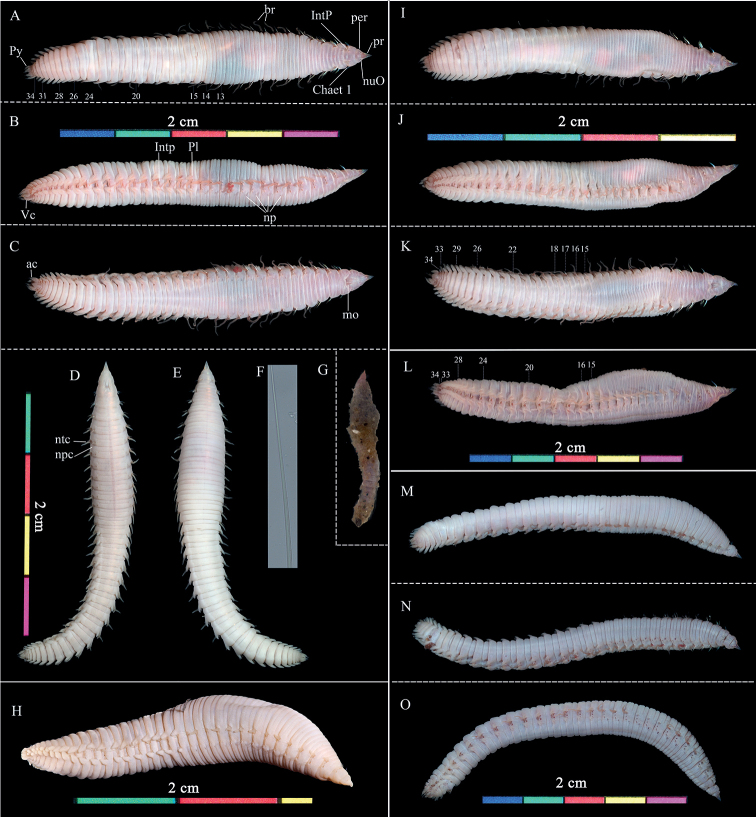
*Travisiaamoyanus* sp. nov. **A–F** holotype (MBM287243) **H–O** paratypes (**H** MBM193597; **I–K** MBM287249; **L** MBM287248; **M–O** MBM287244) **A–C** living specimen in dorsal, lateral, and ventral view, respectively **D** fixed specimen in ventral view **E** same, in dorsal view **F** detail of capillary chaeta **G** tube **H** fixed specimen in lateral view **I–K** alive, in dorsal, lateral, and ventral views, respectively **L** alive, in lateral view **M–O** alive, dorsal, lateral, and ventral views, respectively. Abbreviations: pr, prostomium; per, peristomium; nuO, nuchal organ; mo, mouth; br, branchiae; chaet, chaetiger; IntP, interramal papilla; Pl, parapodia lateral lappet; np, nephridial pores; ntc, notochaetae; npc, neurochaetae; Py, pygidium; ac, anal cirri; Vc, Pygidial ventralmost cirrus.

Preserved specimens white to grey, and living specimens reddish (Fig. 4D, F, G). Body length 18.0–45.0 mm (holotype, 30.0 mm) and 2.0–5.6 (holotype, 3.0 mm) width at widest segment. Prostomium conical, distally pointed. Eyes and prostomial processes absent (Fig. 4A–C, H–N). Peristomium with a pair of nuchal organs (Fig. 4A, H, L). Mouth opening between chaetiger 1 and 2 (Fig. 4C, D, M). Body surface with fine papillae except the distal part of prostomium and branchiae (Fig. 4A–E, G–M).

Branchiae simple, cirriform with 27–31 pairs (holotype: 31 pairs on the left side, 30 pairs on the right side), from chaetigers 2 to chaetigers 28–32. In preserved specimens, branchiae length nearly uniform except for chaetiger 2 and about the last 10 chaetigers.

**Figure 5.**
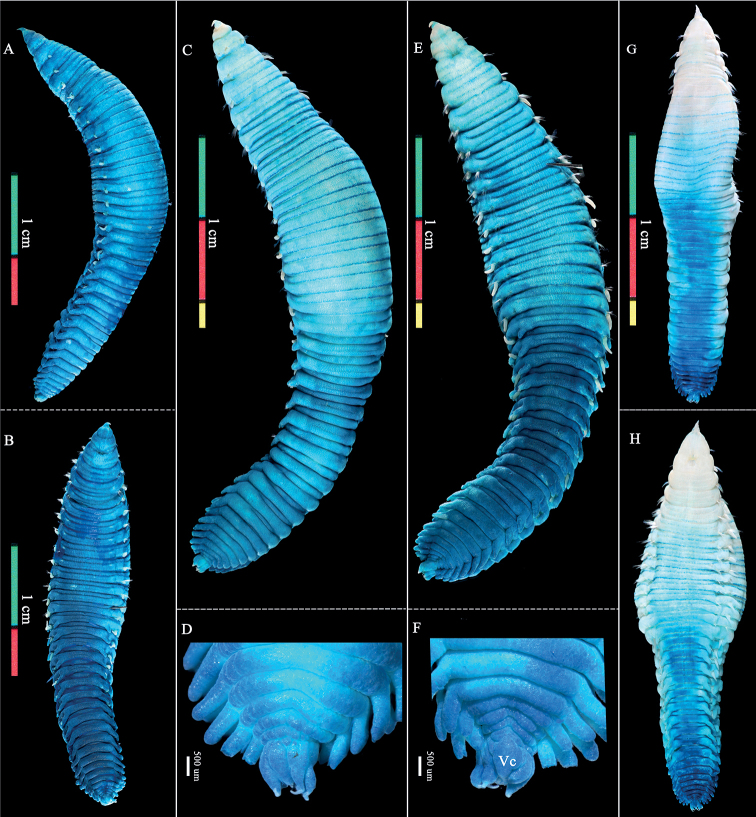
*Travisiaamoyanus* sp. nov., stained with methyl green. Paratypes (**A, B** MBM286089-spec.1; **C–F** MBM286089-spec.2) and non-type specimen (**G, H** MBM286088-Spec.1) **A, C, G** whole body in dorsal view **B, E, H** whole body in ventral view **D** posterior end in dorsal view **F** posterior region in ventral view Abbreviations: Vc, Pygidial ventralmost cirrus.

Body with 34 or 35 segments and corresponding 33 or 34 chaetigers. All chaetae capillary, with a narrow wing (limbate) at one side (Fig. 5 F).

Parapodia biramous, without pre- and postchaetal lobes, notopodial and neuropodial chaetal rami well separated except the posterior end. Interramal pores or lateral sense organs between notopodial and neuropiodial chaetal rami from chaetiger 1 to every succeeding segment, except that occasionally hidden or absent on segment 34 or 35.

Prominent parapodia lateral lappets from chaetiger 15, well developed. Notopodial lobes (lappets) above the bundle of notochaetae. Neuropodial lobes below neurochaetae but missing on last one or two chaetigers. Notopodial and neuropodial lobes triangular except toward the anus, where they become longer and more cylindrical.

Nephridial pores present on chaetigers 3–14, anterior and posterior pores smaller than middle ones. First chaetiger biannulate, chaetigers 2–19 triannulate ventrally and dorsally, chaetigers 20–27 biannulate, 28–34 (35) segments uniannulate. Posterior margin of the last seven or eight segments with more or less obvious crenulations dorsally. Midventral groove absent, if have, present from last four segments (Fig. 4D).

Pygidium as long as about last three segments with a larger triangular mid-ventral process and six lobes. Inner anus with many cirriform papillae.

###### MG staining pattern.

The body surface of specimens has a distinctive staining pattern: the posterior part of the first and the third ring of chaetigers 2–14 show significant staining; from chaetigers 15 to the posterior end the body is deeply stained (Fig. 5).

###### Variations.

Morphological comparison of 23 specimens is provided (Suppl. material 1: table S5). Maximum length ranged from 1.8 to 4.5 mm. Branchiae distribution is frequently asymmetrical on both sides of the body, most specimens have a narrow range (*N* = ±1), except MBM286089-spec.3 (28 pairs on left, 31 pairs on right).

**Figure 6.**
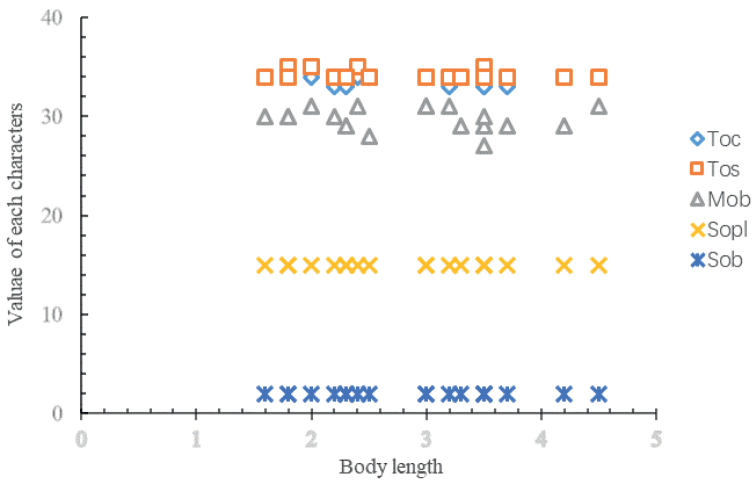
Scatter diagram illustrating variability of five key characters with the body length. Abbreviations: Toc, total number of chaetigers; Tos, total number of segments; Mob, Maximum of branchiae; Sopl, start of parapodial lappets on chaetiger; Sob, start of branchiae.

The maximum number of branchiae ranged from 27–31 pairs among individuals (Fig. 6). Eighteen specimens had 34 segments, and five specimens had 35 segments. Fourteen specimens had 34 chaetigers, and nine specimens had 33 chaetigers.

Body subfusiform in preserved specimens, swollen medially (Fig. 5D, E), while in living specimens, the segments are nearly equal between the prostomium and the anus, usually swollen at the anterior part of the body because of the worm’s peristalsis (Fig. 5A–C, H–N).

###### Type locality.

Coastal waters of Xiamen, China.

###### Etymology.

The specific epithet, *amoyanus*, refers to the type locality of Amoy, the pronunciation of local dialect of Xiamen, a coastal city in Fujian Province, China.

###### Biology.

*Travisiaamoyanus* inhabits sandy sediments from the intertidal to the subtidal (1–2 m depth). It can be strongly malodorous, and the body surface is covered by a viscous mucus tube with sand grains adhering (Fig. 5F).

###### Remarks.

*Travisiaamoyanus* sp. nov. clearly differs from *T.chinensis* in the total number of segments and chaetigers, the beginning of parapodial lappets, and the shape of pygidium. In *T.amoyanus* (34 or 35 segments, 33 or 34 chaetigers), parapodial lappets start from chaetiger 15 and the pygidium with a large triangular mid-ventral process, whereas in *T.chinensis* (30 segments, 29 chaetigers), neuropodial lappets start from chaetiger 16 and notopodial lappets from chaetiger 19 and the pygidium bears no large triangular mid-ventral lobe.

*Travisiaamoyanus* sp. nov. resembles several species in having a similar number of segments and chaetigers (35–36), such as *T.concinna* (Kinberg, 1866) (35 segments and chaetigers) from South Africa, *T.arborifera* (36 chaetigers) from Indian Ocean, and *T.filamentosa* (35–36 segments, 35 chaetigers) from California. However, *T.amoyanus* sp. nov. can be distinguished from *T.arborifera* and *T.filamentosa* by having cirriform branchiae, the latter two species have branched branchiae. *Travisiaamoyanus* sp. nov. differs still from *T.concinna* in having 31 (vs 33) pairs of branchiae, and parapodial lappets starting from chaetiger 15 (vs 17 or 18). In addition, *T.amoyanus* sp. nov. has 31 pairs of branchiae and parapodial lappets from chaetigers 15, while *T.fusiformis* Kudenov, 1975 has 34 pairs of branchiae, notopodial lappets from chaetigers 2 and neuropodial lappets from chaetiger 17.

*Travisiaamoyanus* sp. nov. is much closer to *T.japonica* Fujiwara, 1933 from Japan and *T.gigas* Hartman, 1938 from California in the starting segments of parapodial lappets. But, the new species and *T.gigas* can be distinguished in the following aspects: (1) 34 or 35 segments and 33 or 34 chaetigers in *T.amoyanus*, 46 segments and 46 chaetigers in *T.gigas*; (2) 31 pairs of branchiae in *T.amoyanus*, 44 pairs in *T.gigas*; (3) pygidium with a large triangular mid-ventral process and six cylindrical lobes in *T.amoyanus*, without triangular mid-ventral process in *T.gigas*.

*Travisiajaponica* is considered to have a wide-ranging body segment count (32–43 segments), and the species has been recorded from a wide range of geographic regions (Dauvin and Bellan 1994). However, Fujiwara (1933) stated explicitly that *T.japonica* has a relatively fixed number of segments (39, seldom 40) based on examination of a considerable number of specimens. Therefore, in this comparison, we used the original description data and suggest that records of *T.japonica* from non-Japanese areas need to be re-evaluated and might represent potentially undescribed species.

*Travisiaamoyanus* sp. nov. is distinguishable from *T.japonica* by the following characters: the number of segments (34 or 35 in *T.amoyanus* vs 39 or 40 in *T.japonica*), the number of chaetigers (33 or 34 in *T.amoyanus* vs 39 or 40 in *T.japonica*), the number of branchiae (27–31 pairs in *T.amoyanus* vs 25 pairs in *T.japonica*), the distribution of interramal pores (1–33 or 34 chaetigers in *T.amoyanus* vs 1–29 chaetigers in *T.japonica*), the number of nephridial pores (12 pairs in *T.amoyanus* vs 11 pairs in *T.japonica*). In fact, the difference between these two species also had been noticed by Monro (1934: p374): “*T.japonica* Fujiwara is close to *T.chinensis* (regarded herein as *T.amoyanus*), but has 39 to 40 chaetigers”.

###### Distribution.

Currently only found from Xiamen coastal waters.

## Supplementary Material

XML Treatment for Travisia

XML Treatment for Travisia chinensis

XML Treatment for Travisia amoyanus

## Supplementary materials

Supplementary material 1 Tables Data type Morphological, phylogenetic. Explanation note **Tables: table S1**: Sampling information and museum registration numbers of *Travisiaamoyanus* sp. nov.; **table S2**: Primers and annealing temperatures used in this study; **table S3**: Information on taxa and sequences used in phylogenetic analyses; **table S4**: Pairwise distances in 18S fragment within and among species of *Travsiva* based on Kimura 2-parameter (the lower-left corner) and p-distance model (the upper right corner); **table S5**: Main morphological characters of studied specimens of *Travisiaamoyanus* sp. nov. https://binary.pensoft.net/file/765771This dataset is made available under the Open Database License (http://opendatacommons.org/licenses/odbl/1.0/). The Open Database License (ODbL) is a license agreement intended to allow users to freely share, modify, and use this Dataset while maintaining this same freedom for others, provided that the original source and author(s) are credited.Deyuan Yang, Xuwen Wu, Zhi Wang, Xiaoyu Zhao, Jiangshiou Hwang, Lizhe Cai

Supplementary material 2 ML tree based on 16S and 18S Data type Phylogenetic. https://binary.pensoft.net/file/765772This dataset is made available under the Open Database License (http://opendatacommons.org/licenses/odbl/1.0/). The Open Database License (ODbL) is a license agreement intended to allow users to freely share, modify, and use this Dataset while maintaining this same freedom for others, provided that the original source and author(s) are credited.Deyuan Yang, Xuwen Wu, Zhi Wang, Xiaoyu Zhao, Jiangshiou Hwang, Lizhe Cai

## References

[B1] Annenkova NP (1937) Polychaete fauna of the northern part of the Japan Sea. Issledovaniya fauny morei. Zoologicheskii Institut Akademii Nauk USSR Explorations des Mers de l’URSS 23: 139–216. [In Russian]

[B2] Augener H (1922) Revision der australischen Polychaeten-Typen von Kinberg. Arkiv för Zoologi 14(8): 1–42. 10.5962/bhl.part.7728

[B3] Blake JA Maciolek NJ (2020) Travisiidae Hartmann-Schröder, 1971, new family status. In: Blake JA Maciolek NJ (Eds) Handbook of Zoology.Annelida. Volume 2: Pleistoannelida, Sedentaria II. Walter de Gruyter, Berlin, 302–311. 10.1515/9783110291681-009

[B4] Dauvin JC Bellan G (1994) Systematics, ecology and biogeographical relationships in the family Travisiinae (Polychaeta, Ophelidae). Mémoires du Muséum National d’Histoire Naturelle 162: 169–184.

[B5] Fauvel P (1932) AnnelidaPolychaeta of the Indian Museum. Calcutta Memoirs of the Indian Museum 12(1): 1–262.

[B6] Fujiwara T (1933) On a new species of Japanese Polychaeta, *Travisiajaponica* sp. nov. Journal of Science of the Hiroshima University, Series B, Division 1 (Zoology) 2: 91–103.

[B7] Glover A Dahlgren T Wiklund H Mohrbeck I Smith C (2016) An end-to-end DNA taxonomy methodology for benthic biodiversity survey in the Clarion-Clipperton Zone, Central Pacific Abyss. Journal of Marine Science and Engineering 4(1): e2. 10.3390/jmse4010002

[B8] Grube AE (1869) Familie der Opheliaceen. Schlesische gesellschaft für vaterlandische kultur Breslau Jahresbericht 46: 59–68.

[B9] Hartman O (1938) Descriptions of new species and new generic records of polychaetous annelids from California of the families Glyceridae, Eunicidae, Stauronereidae and Opheliidae. University of California Publications in Zoology 43: 93–111.

[B10] Hartman O (1969) Atlas of the sedentariate polychaetous annelids from California. Allan Hancock Foundation, University of Southern California, Los Angeles, 812 pp.

[B11] Johnston G (1840) Miscellanea Zoologica. British annelids. Annals and Magazine for Natural History London 1(4): 368–375. 10.1080/00222934009512507

[B12] Kalyaanamoorthy S Minh BQ Wong TKF von Haeseler A Jermiin LS (2017) Model Finder: Fast model selection for accurate phylogenetic estimates. Nature Methods 14(6): 587–589. 10.1038/nmeth.4285 28481363 PMC5453245

[B13] Katoh K Rozewicki J Yamada KD (2019) MAFFT online service: Multiple sequence alignment, interactive sequence choice and visualization. Briefings in Bioinformatics 20(4): 1160–1166. 10.1093/bib/bbx108 28968734 PMC6781576

[B14] Kobayashi G Kojima S (2021) *Travisiasanrikuensis*, a new species of Travisiidae (Annelida) from the Lower Bathyal Zone of the Northwestern Pacific. Species Diversity : An International Journal for Taxonomy, Systematics, Speciation, Biogeography, and Life History Research of Animals 26(2): 131–136. 10.12782/specdiv.26.131

[B15] Kudenov JD (1975) Sedentary polychaetes from the Gulf of California. Journal of Natural History 9(2): 205–231. 10.1080/00222937500770131

[B16] Kükenthal W (1887) Die Opheliaceen der Expedition der “Vettore Pisani”. Jenaische Zeitschrift für Naturwissenschaft 21: 361–373. https://www.biodiversitylibrary.org/page/8612269

[B17] Kumar S Stecher G Li M Knyaz C Tamura K (2018) MEGA X: Molecular Evolutionary Genetics Analysis across computing platforms. Molecular Biology and Evolution 35(6): 1547–1549. 10.1093/molbev/msy096 29722887 PMC5967553

[B18] Salazar-Vallejo SI Carrera-Parra LF Muir AI de Léon-González JA Piotrowski C Sato M (2014) Polychaete species (Annelida) described from the Philippine and China Seas. Zootaxa 3842(1): 1–68. 10.11646/zootaxa.3842.1.1 25082164

[B19] Law CJ Dorgan KM Rouse GW (2014) Relating divergence in polychaete musculature to different burrowing behaviors: A study using Opheliidae (Annelida). Journal of Morphology 42: 548–571. 10.1002/jmor.20237 24435812

[B20] Maciolek NJ Blake JA (2006) Opheliidae (Polychaeta) collected by the R/V Hero and the USNS Eltanin cruises from the Southern Ocean and South America. Scientia Marina 70(S3): 101–113. 10.3989/scimar.2006.70s3101

[B21] Maekawa N Hayashi I (1999) Taxonomic study on the genus *Onuphis* (Polychaeta, Onuphidae) from Japan and adjacent seas, with descriptions of six new species. Bulletin of the National Science Museum, Tokyo, Series A 25: 163–214.

[B22] Monro CCA (1934) On a collection of Polychaeta from the coast of China. Annals and Magazine of Natural History (Series 10) 13: 353–380. 10.1080/00222933408654824

[B23] Moore JP (1923) The polychaetous annelids dredged by the U.S.S. “Albatross” off the coast of southern California in 1904. IV. Spionidaeto Sabellariidae. Proceedings. Academy of Natural Sciences of Philadelphia 75: 179–259.

[B24] Nguyen LT Schmidt HA von Haeseler A Minh BQ (2015) IQ-TREE: A fast and effectivestochastic algorithm for estimating maximum-likelihood phylogenies. Molecular Biology and Evolution 32(1): 268–274. 10.1093/molbev/msu300 25371430 PMC4271533

[B25] Read G Fauchald K [Eds.] (2022) World Polychaeta Database. *Travisia* Johnston, 1840. World Register of Marine Species. https://www.marinespecies.org/aphia.php?p=taxdetails&id=129417 [Accessed on 2022-06-26]

[B26] Rizzo A Salazar-Vallejo SI (2020) A new species of *Travisia* (Annelida, Travisiidae) from Campos Basin, Brazil. Studies on Neotropical Fauna and Environment 56(1): 1–9. 10.1080/01650521.2020.1752512

[B27] Ronquist F Teslenko M van der Mark P Ayres D Darling A Hohna S Larget B Liu L Suchard M Huelsenbeck J (2012) MrBayes 3.2: Efficient Bayesian phylogenetic inference and model choice across a large model space. Systematic Biology 61(3): 539–542. 10.1093/sysbio/sys029 22357727 PMC3329765

[B28] Talavera G Castresana J (2007) Improvement of phylogenies after removing divergent and ambiguously aligned blocks from protein sequence alignments. Systematic Biology 56(4): 564–577. 10.1080/10635150701472164 17654362

[B29] Wiklund H Neal L Glover AG Drennan R Muriel R Dahlgren TG (2019) Abyssal fauna of polymetallic nodule exploration areas, eastern Clarion-Clipperton Zone, central Pacific Ocean: Annelida: Capitellidae, Opheliidae, Scalibregmatidae, and Travisiidae. ZooKeys 883: 1–82. 10.3897/zookeys.883.36193 31719773 PMC6828828

[B30] Yang D Sun R (1988) Polychaetous annelids commonly seen from Chinese waters. China Agriculture Press, Beijing, 352 pp. [in Chinese]

[B31] Zhang D Gao F Jakovlić I Zou H Zhang J Li WX Wang GT (2020) PhyloSuite: An integrated and scalable desktop platform for streamlined molecular sequence data management and evolutionary phylogenetics studies. Molecular Ecology Resources 20(1): 348–355. 10.1111/1755-0998.13096 31599058

