# Supplementary material for: ﻿Redescription of a rarely encountered species Travisachinensis Grube, 1869 (Annelida, Travisiidae), including a description of a new species of Travisa from Amoy, China
Source: Zookeys. 2022 Nov 4;1128:1–17. doi: 10.3897/zookeys.1128.90020 (PMC9836580; doi:10.3897/zookeys.1128.90020)
Supplement: Supplementary material 2 — ML tree based on 16S and 18S [file zookeys-1128-001_article-90020__-s002.docx]

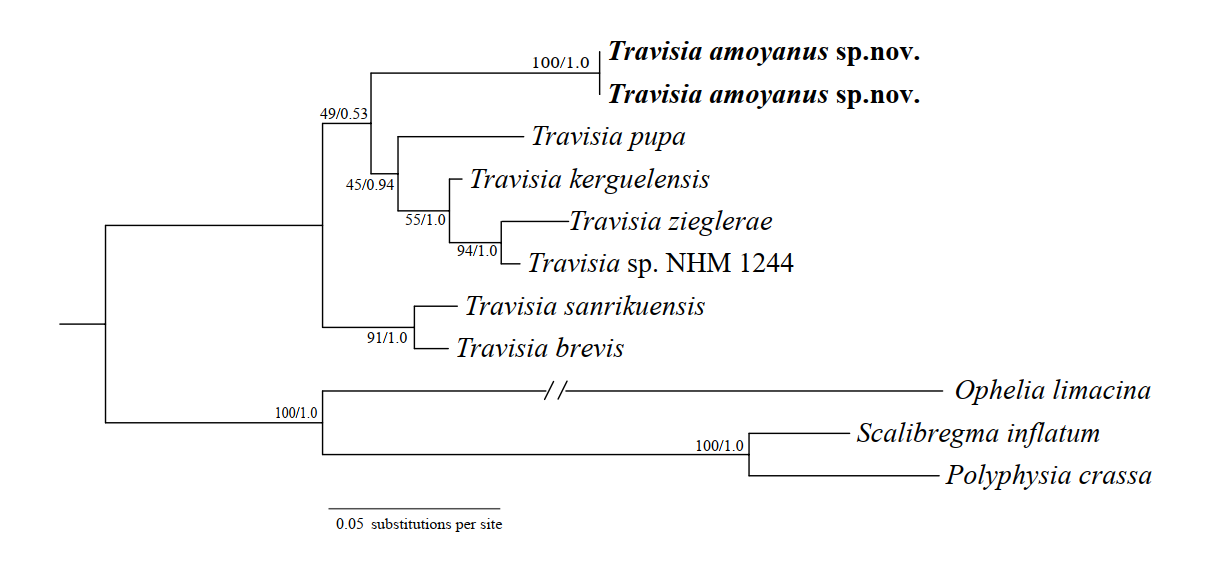


Figure S1. Maximum-likelihood (ML) phylogenetic tree of Travisiidae based on 16S and 18S sequences under GTR+F+G4 model (18S) and TN+F+I model (16S), GTR+G model (16S) and TN+F+I model (18S) for BI analysis. Support values were based on 300000 ultrafast bootstraps from maximum likelihood (left) and posterior probability values from Bayesian analyses (right). *Travisia amoyanus* sp. nov. is shown in bold type.
